# Differences in Vaginal Lactobacilli Composition of Iranian Healthy and Bacterial Vaginosis Infected Women: A Comparative Analysis of Their Cytotoxic Effects with Commercial Vaginal Probiotics

**DOI:** 10.5812/ircmj.3533

**Published:** 2013-03-05

**Authors:** Elahe Motevaseli, Mahdieh Shirzad, Reza Raoofian, Seyyed-Mohammad Hasheminasab, Maryam Hatami, Mehdi Dianatpour, Mohammad-Hossein Modarressi

**Affiliations:** 1 Medical Genetics Department, Tehran University of Medical Sciences, Tehran, IR Iran

**Keywords:** Vagina, Bacterial Vaginosis, Women, T-Lymphocytes, Cytotoxic, Probiotic, Iran

## Abstract

**Background:**

Vaginal flora of healthy women is dominated by Lactobacillus species which can prevent bacterial vaginosis.

**Objectives:**

The current study aimed to determine the differences in vaginal lactobacilli composition of Iranian healthy and bacterial vaginosis (BV) infected women and compared their cytotoxic effects with commercial vaginal probiotics.

**Patients and Methods:**

One hundred and seventy eight vaginal specimens were collected from healthy and BV infected women. Lactobacillus colonies were obtained by culturing on laked blood BHI and MRS medias and genetically defined by 16s rRNA sequencing. Differentiating the specimens to normal, intermediate and BV infected were carried out by Ison and Hey grading protocol. Identification of Lactobacillus strains in vaginal specimens were performed by Multiplex PCR. The inhibitory effects of lactobacilli on Hela (tumoral cervical cells) and HNCF-pi52 (normal cervical cells) were conducted by MTT and trypan blue assays.

**Results:**

L. crispatus, L. gasseri, L. iners, L. jensenii, L. acidophilus and L. rhamnosus were the most frequently occurring species in vagina of healthy Iranian women. L. crispatus and L. jensenni were significantly higher in the normal than in the BV infected groups. Also the cytotoxic effect of L. crispatus on tumoral cervical cells was higher than other lactobacilli including commercial probiotics.

**Conclusions:**

As L. crispatus and L. jensenni were significantly higher in BV infected women and the cytotoxic effect of L. crispatus on tumoral cervical cells was high, introduction of new probiotics seems necessary.

## 1. Background

The healthy human vagina is physiologically dominated by Lactobacillus species, which are responsible for protecting the host from bacterial infections (1). Lactobacilli belong to a group of beneficial organisms termed probiotics. Probiotics are defined as live micro-organisms which confer a health benefit on the host when administered in adequate amounts (2).Probiotics have been widely used to conserve the normal flora of gastrointestinal system and female external genitalia (3).Nowadays, they are used for treatment of clinical situations such as acute diarrhea, inflammatory bowel disease, and a number of vaginal infections such as candidiasis and bacterial vaginosis (BV) (4-7). In order to design improved probiotics, better understanding of each population’s normal vaginal flora seems necessary. The microbiota of female external genitalia has been studied in a number of studies and various results have been reported from different populations (8, 9). Some studies indicated that *L. crispatus*, *L. jensenii*, *L. gasseri* and *L. iners* were predominant in normal vaginal microflora (10) but other studies reported different results (11, 12).Meanwhile, the composition of vaginal lactobacilli in healthy and infected women were examined in order to determine which lactobacillus species may prevent bacterial vaginosis efficiently (1, 13, 14).Further it was shown that there were specific species which were more common in healthy individuals and it is important to investigate their different effects.Recently, anticancer effects of Lactobacillus species have been discussed. Some studies investigated Lactobacillus effects on colon and gastric cancer cell lines (15, 16). Various mechanisms were reported for their anticancer effects on colon and gastric cancers, including stimulation and modulation of immunity, reduction of potential carcinogens, regulation of cytokines and modulation of the intermediate biomarkers of carcinogenesis (17, 18). Also, their potential use as an adjuvant therapy in cancer treatment was analyzed and it was demonstrated that they increase the sensitivity of cancer cells to 5-fluorouracil (19).Moreover, vaginal lactobacilli are also colonizing cervical area, so they can play an important role in maintaining cervical physiological conditions such as cancer prevention. Cervical cancer is the most frequently diagnosed female cancer in developing countries and the second leading cause of cancer death in women worldwide (20, 21). Although, the role of Human Papilloma Virus (HPV) in cervical carcinogenicity has been established in recent years and HPV infection has a high prevalence, but the incidence of cervical neoplastic is less than what is expected from prevalence of HPV infection.It has been revealed that most of the HPV infections resolve spontaneously, but the reason has still remained unknown. Possibly, other factors such as environment and genetics play a role in tumor formation and prevention. Of these environmental factors, vaginal lactobacilli of female genitalia should be considered precisely. A recent study investigated the different component effects of *L. crispatus* and *L. gasseri* on cervical cells and demonstrated that the supernatants of these lactobacilli had more cytotoxic effects on tumoral cervical cells compared to those of normal cells (unpublished data). These bacteria are responsible for many physiologic and modulatory activities in that area, thus it was decided to investigate the effect of other lactobacillus strains present in female genitalia. First the prevalence of vaginal lactobacilli in normal and BV infected Iranian women was investigated, and then the anti-proliferative effects of vaginal lactobacilli isolated from healthy Iranian women with currently used vaginal probiotics were analyzed. HeLa cell was used as a cervical cancer cell line and PI 52 (HNCF) cells as a normal cervical cell line to compare lactobacilli effects on normal and tumoral cells.

## 2. Objectives

The current study aimed to determine the differences in vaginal lactobacilli composition of Iranian healthy and bacterial vaginosis (BV) infected women and compared their cytotoxic effects with commercial vaginal probiotics

## 3. Patients and Methods

### 3.1. Study Population, Sample Collection and Media Condition

Women were recruited at Gynecology Outpatient Clinic of Imam Khomeini Hospital affiliated to Tehran University of Medical Sciences in Tehran, Iran between February 2009 and February 2011. All women were not pregnant, of premenopausal reproductive age, ranging from 18 to 45 years (mean 31.5 ± 5.9 years). They had not taken any antibiotic or antimycotic compounds, vaginal medications or suppositories or contraceptive spermicides in the past 30 days. Pregnant and menopause women were excluded because it was anticipated that their vaginal flora might differ substantially from that of other women attending the clinic. After obtaining informed consent, each participant completed a questionnaire on menstruating regularly (25- to 35-day menstrual cycles), yogurt consumption (low ≤ 100 ml/day, medium between 100 and 250 ml/day, high ≥ 250 ml/day) and reproductive and sexual health history including vaginal discharge. Three sterile swabs were inserted into the women vagina through a speculum and specimens of vaginal fluid were obtained by brushing the posterior vaginal fornix and exocervix. Swabs were carefully removed to prevent contamination with micro flora of the vulva and vaginal orifice. One of them was used for gram staining procedure and the second swab from each woman was suspended in 3 ml laked blood BHI broth (because *L. inerscannot* grow in MRS broth). This new medium was applied in this research for the first time and was prepared by a simple method. Firstly defibrinated sheep blood was lysed through five freeze and thaw cycles and then the lysate was centrifuged to remove the red blood cell debris. At last, 5% lakedblood was added to 3 ml sterilized BHI broth to make laked blood BHI broth. The last swab was used to culture on MRS agar directly. Two hours after sample collection, swabs were removed from the cultures, and then the cultures were vortexed for 20 seconds. One milliliter of each specimen was used for mixing DNA extraction and the remaining was incubated in 37ºC for 48 hours.

### 3.2. Gram Staining and Grading of Slides

One of the swabs was enrolled onto a glass slide, which was then air-dried, heat-fixed, and Gram-stained.Smears were graded according to method described by Ison(22)as grade 1 (normal flora), Lactobacillus morphotype only; grade 2 (intermediate flora), reduced lactobacillus morphotype with mixed bacterial morphotypes and grade 3 (BV), mixed bacterial morphotypes with few or absent lactobacillus morphotypes.

### 3.3. Bacterial Colony Isolation and Biochemical Identification

The cultures were diluted in phosphate-buffered saline (pH 7) and 0.1 ml of each dilution was spread on MRS agar and laked blood BHI agar (+1.5% agar) plates. Then, the plates were incubated in candle-jar at 37º C for 3 days. The colonies grown on MRS and laked blood BHI agar plates were examined for their gram reaction and morphology. Colonies appearing gram positive and catalase negative were subjected to biochemical reactions and identified by comparing their sugar fermentation patterns with the scheme described in Bergy's manual of systematic bacteriology. A total of 120 gram positive, catalase negative and rod shaped lactic acid bacteria isolates were obtained from laked blood BHI agar and MRS agar identified as lactobacilli and their DNA were extracted for molecular species identification.

### 3.4. Culture preparation and Colony Formation Unit (CFU) Adjustment

From colonies which were identified (biochemically and genetically) as Lactobacillus strains, three species from healthy women were selected for cytotoxicity evaluation on normal and tumoral cervical cells and a comparative analysis of their growth inhibitory effect with three commercial vaginal probiotics was performed. These six species were selected for further evaluation and the comparison of cytotoxity was undertaken for *L. crispatus* strain SJ-3C-US (LbC), *Lactobacillus rhamnosus* GG, *Lactobacillus paracasei* subsp. paracasei ATCC 25302 from vaginal isolates and *Lactobacillus casei* var. Rhamnosusdoderlein (Gynophiluslyocentre), *Lactobacillus acidophilus* NCFM (probioti-NCF) and *Lactobacillus helveticus* LA 401 *candisis* (Lactibiancecandisis 10M) from commercial vaginal probiotics. Each lactobacillus was inoculated in 50 ml MRS broth and incubated at 37?C for 24 hours. Using 500 µl of the cultured lactobacilli, colony formation unit (CFU) was determined through serial dilution and Miles and Misra surface colony count protocol on MRS agar. The CFU of all cultures was adjusted to the same number of 109 CFU per milliliter. Then, the supernatant of the cultures was used for cytotoxity assays.

### 3.5. Culture Supernatant Preparation

For supernatant preparation, the cultures were centrifuged at 5,000 g for 15 min at 4°C. The resulting lactobacilli supernatants (LS) were filtered through a 0.2 μm membrane filter to remove the remaining bacteria and debris. The pH of LS was determined by pH meter. As pH of 5 lactobacilli supernatants was 4, to examine if lactic acid produced by the lactobacilli and pH change would affect the tests, non-cultured MRS broth adjusting to pH of 4 with HCl and/or lactate was used in co-culture tests [MRS of pH = 4 with HCL (MRH), MRS of pH = 4 with lactic acid (MRL)].

### 3.6. DNA Extraction

As already mentioned, one milliliter of laked blood BHI broth was used for DNA extraction. The specimen was centrifuged at 14,000 rpm for 5 min. The pellet’s DNA was extracted and purified with a DNG plus kit (Sinaclon, Iran) according to the manufacturer's instructions, resulting in 50 μl of DNA solution. DNA extracts were stored at –20°C and were used for the species specific PCR experiments. The DNA of the isolated Lactobacillus colonies was also extracted by this protocol.

### 3.7. PCR Protocols and Conditions

The PCR mixtures consisted of PCR buffer with 1.5 mM of MgCl2, 10 pmol of each primer, 2.0 μM of each deoxyribonucleoside triphosphate, 0.1 μl of Taq DNA polymerase, and 1.5 μl of template DNA solution in a final volume of 25 μl. PCR reaction condition were as follows: Predenature at 95°C for 3 minutes, melt at 95°C for 30 seconds; annealing at 54–64°C (depending on each procedure) for 30 seconds; extension at 72°C for 20-120 seconds (depending on each procedure); 35 cycles; a final extension at 72°C for 7 minutes. All PCR reactions were performed by ABI (7300, America) instrument. PCR primers were indicated in Table 1. The PCR products are visualized after electrophoresis in 2% agarose gel and stained with ethidium bromide.

**Table 1.** PCR Primers

	Base Pairs	Forward Primer	Reverse Primer
**Universal 16s rRNA**	1500 bp	AGAGTTTGATCTGGCTCAG	AAGGAGGTGATCCAGCC
**Multiplex PCR**			CCTCTTCGCTCGCCGCTACT
Group1	450 bp	ACAGATGGATGGAGAGCAGA	
Group 2	300 bp	ATTGTAGAGCGACCGAGAAG	
Group 3	400 bp	CTAGCGGGTGCGACTTTGTT	
Group 4	350 bp	AAACCGAGAACACCGCGTT	
**Group II**			
*L. acidophilus*	210 bp	TGCAAAGTGGTAGCGTAAGC	CCTTTCCCTCACGGTACTG
*L. crispatus*	522 bp	AGGATATGGAGAGCAGGAAT	CAACTATCTCTTACACTGCC
*L. gasseri*	360 bp	AGCGACCGAGAAGAGAGAGA	TGCTATCGCTTCAAGTGCTT
*L. jensenii*	700 bp	AAGAAGGCACTGAGTACGGA	CCTTTCCCTCACGGTACTG
**Group III**			
*L. paracasei*	312 bp	CTAGCGGGTGCGACTTTGTT	GGCCAGCTATGTATTCACTGA
*L. rhamnosus*	113 bp	CTAGCGGGTGCGACTTTGTT	GCGATGCGAATTTCTATTATT
**Group IV**			
*L. * *salivarius*	411 bp	AATCGCTAAACTCATAACCT	CACTCTCTTTGGCTAATCTT
*L. reuteri*	303 bp	CAGACAATCTTTGATTGTTTAG	GCTTGTTGGTTTGGGCTCTTC
*L. plantarum*	248 bp	ATTCATAGTCTAGTTGGAGGT	CCTGAACTGAGAGAATTTGA
*L. fermentum*	192 bp	ACTAACTTGACTGATCTACGA	TTCACTGCTCAAGTAATCATC
*L. iners*	180 bp	ACAGGGGTAGTAACTGACCTTTG	ATCTAATCTCTTAGACTGGCTATG

Sequencing of 16S rRNA of vaginal lactobacillus colonies was performed according to the methodology described before (23, 24) , universal primers and sequences were compared with the data in Genbank. Multiplex PCR method described before (25)was employed to determine 11 lactobacilli but not *L. inersthus*, specific primers were used for *L. iners* identification.

### 3.8. Cell Culture

The human cervical cancer cell line, Hela and human normal fibroblast-like cervical cell HNCF-PI 52 was obtained from National Cell Bank of Iran, Pasteur Institute of Iran (Tehran, Iran) and were cultured at 37°C with a humidified incubator, 5% CO2, in RPMI 1640 medium containing 10% heat inactivated fetal calf serum, 1% penicillin/streptomycin (50 U/ml penicillin and 50 μg/ml streptomycin) and 1.5% HEPES (Ph=7.2).

### 3.9. Cytotoxicity Assay by MTT and Trypan Blue Staining

MTT (3-[4,5-dimethylthiazol-2-yl](23)-2,5-diphenyl tetrazolium bromide) cytotoxicity assay was carried out according to Denizot and Lang(26).This colorimetric assay was based on the capacity of mitochondria succinate dehydrogenase enzymes in living cells to reduce the yellow water-soluble substrate (MTT) into an insoluble, colored formazan product, which was measured spectrophotometrically. To determine the optimal cell number of cell lines (Hela and HNCF) for MTT assay, 5.10^2^ to 5.10^4^ cells were seeded in 96 well plates and appropriate cell number was detected. For Hela 1.104 cells and for HNCF 5.10^3^ cells were seeded in each well containing 100 μl of standard medium. After overnight growth, cells were treated for 24 h with different concentrations of 7 lactobacilli supernatants and 3 controls; then, the viability of the cells was detected by MTT conversion. The percent inhibition of cell proliferation by the extracts was calculated based on the difference in the inhibitory effect between the treated cell lines and their respective controls, where 100% cell proliferation was taken from the corresponding controls.

Viability (% of control) = [(A sample_A blank) (A control_A blank)-1] × 100

A graph of absorbance against the concentration of the drug was plotted and the inhibitory effect (IC50) was calculated as the drug concentration that was required to reduce the absorbance to half of the control, based on the dose-response curve for the different extracts. Also, cell viability was assessed by means of the trypan blue dye exclusion method (Sigma Chemical Co., St. Louis MO, USA).

### 3.10. Statistical Analysis

Data were analyzed using SPSS software (Version 15, Chicago, IL, USA). Chi-square test was used for analysis of the correlation between qualitative variables. Mann-Whitney test was used to compare IC50 (half maximal inhibitory concentration) of lactobacilli components treated cells to pH and lactic acid modified, and pretreatment of controls. All data were expressed as a mean ± standard error (SE) of three separate experiments, P < 0.05 was considered as statistically significant.

## 4. Results

### 4.1. Hey Grading of Specimens

All samples were graded according to modified Ison and Hay criteria and divided into normal grade 1 (n=54), intermediate grade 2 (n=60), and BV groups grade 3 (n=64). There were no statistically significant differences in the mean age, among the three groups. Among all of the clinical and social information collected by the questioners, some points seemed to be more noticeable. The first point was that amounts of yogurt consumption were not significantly different among all grades. The second point was that the vaginal discharge rate in grade 3 was higher than other groups (P < 0.05) which confirmed the grading method. The third point was that menstrual irregularity rate was higher in infected women but it was not significant (P = 0.1).

### 4.2. Bacteria Isolated on Media

**Figure 1.**
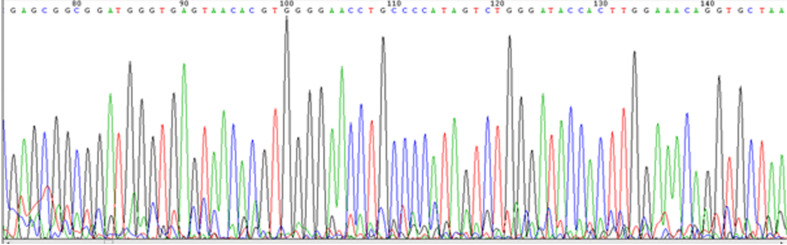
Sequencing graph of L.crispatus 16S rRNA Gene

A total of 120 gram positive, catalase negative and rod shaped lactic acid bacteria isolates were obtained and were tentatively identified as belonging to the Lactobacillus genus. All isolates were shown to be Lactobacillus using 16S rRNA sequencing (Figure 1).

### 4.3. Detection Rate of Lactobacilli in Three Groups by PCR

The results of PCR assay for 178 samples were shown in Table 2.

**Table 2.** The Prevalenceof Lactobacilli in Different Grades

	Grade 1, No. (%)	Grade 2, No. (%)	Grade 3, No. (%)
**Multiplex PCR group I**	0 (0)	0 (0)	0 (0)
*Lactobacillus delbrueckii*	0 (0)	0 (0)	0 (0)
**MultiplexPCR group II**	48 (88.9)	46 (76.7)	46 (71.9)
*Lactobacillus acidophilus*	16 (29.6)	4 (6.7)	10 (15.6)
*Lactobacillus crispatus*	36 (66.7)	16 (26.7)	24 (37.5)
*Lactobacillus gasseri*	16 (29.6)	6 (10)	14 (21.9)
*Lactobacillus jensenii*	16 (29.6)	14 (23.3)	6 (9.4)
**Multiplex PCR group III**	24 (44.4)	38 (63.3)	46 (71.9)
*Lactobacillus paracasei*	10 (18.5)	10 (16.7)	8 (12.5)
*Lactobacillus rhamnosus*	16 (29.6)	24 (40)	30 (46.9)
**Multiplex PCR group IV**	14 (25.9)	8 (13.3)	14 (21.9)
*L. salivarius*	2 (3.7)	2 (3.3)	4 (6.3)
*L. reuteri*	2 (3.7)	2 (3.3)	2 (3.1)
*L. plantarum*	6 (11.1)	4 (6.7)	4 (6.3)
*L. fermentum*	6 (11.1)	2 (3.3)	6 (9.4)
*L. iners*	30 (55)	33(55)	50 (83)

**Figure 2.**
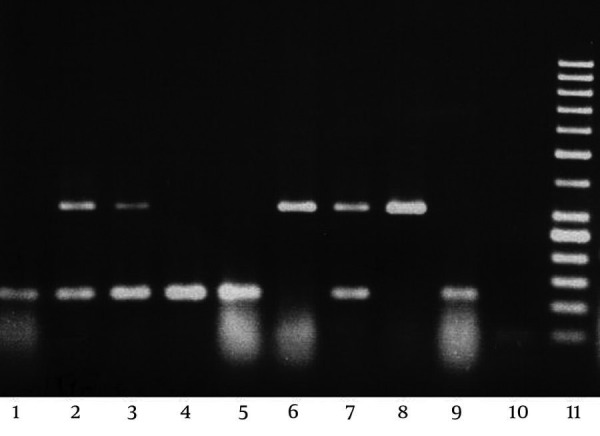
PCR group III, L. paracasei band: 312 bp, L. rhamnosus: 113 bp lane 1-9: samples lane 10: negative control lane 11: 50 bp ladder

One sample gel electrophoresis of PCR products was shown in Figure 2. Using the combined results, *L. crispatus*, *L. gasseri*, *L. iners*, *L. jensenii*, *L.acidophilus* and *L. rhamnosus* were the most frequently occurring species in the healthy vaginas of Iranian women. The detection rate of L. crispatus, was significantly higher in the normal group (grade 1) than in the intermediate (grade 2) and BV (grade 3) group (P =0.007) and *L. jensenni* was higher in normal group than BV group (P = 0.047).Group specific PCR indicated that group II lactobacilli were mostly common in all grades and although they had higher prevalence in grade I but it was not significant. No isolate belonged to group I (Table 2). Group III lactobacilli was higher in BV group than normal group (P = 0.033). *L. rhamnosus* and *L. iners* were higher in BV group than the others but it was not statistically significant.

### 4.4. Cytotoxicity Assay

**Figure 3.**
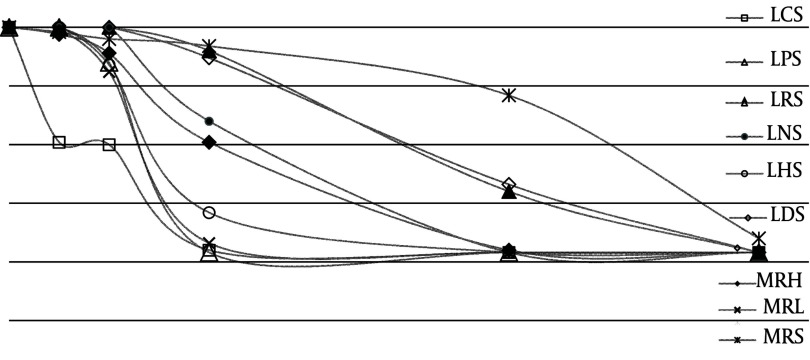
Cytotoxicity Activity of Different Lactobacilli Supernatants and Controls onHela cells. LCS: L. crispatus supernatant, LPS: L. paracasei supernatant, LRS: L. rhamnosus supernatant, LNS: L. acidophilus NCFM supernatant, LHS: L. helveticus supernatant, LDS: L. casei, MRH: MRS with HCl, MRL: MRS with lactic acid, MRS

First, HeLa cells were exposed to the different concentrations of lactobacilli culture supernatants to investigate their cytotoxic effects. MTT assays revealed that after incubation with HeLa cells for 24 hours, each of these supernatants had a dose-dependent inhibitory effect on HeLa cell viability (Figure 3). Of these compounds, *L. crispatus* supernatant reduced cell viability to the greatest extent.

Then to compare their cytotoxic effects on tumoral and normal cells, HNCF cells were used as a representative of normal cervical cells. The prominent anti-proliferative effect of the isolated vaginal lactobacilli, the commercial vaginal probiotics and pH, lactic acid and media controls on HeLa and HNCF cell lines, as revealed by its IC50 based on the MTT assay, were shown in Table 3.

**Table 3.** The IC50 of Lactobacilli Supernatants on Hela and HNCF Cells.

Lactobacillus Strain	pH	IC50 of Culture supernatant (V) /total media(V), %	
		Hela	HNCF
**Culture supernatants (vaginal lactobacilli)**			
*L. crispatusstrain*SJ-3C-US	4	4	14
*L.paracasei* subsp. paracasei ATCC 25302	4	14	14
*L. rhamnosus* GG	5	40	35
**Culture supernatants (commercial vaginal probiotics)**			
*L. acidophilus* NCFM	4	25	14
*L. acidophilluscandisis*	4	16	14
*L. casei* var. Rhamnosusdoderlein	5	40	35
**Controls**			
MRH (MRS with HCL)	4	20	32
MRL (MRS with lactic acid)	4	14	14
MRS	6.5	58	55

The IC50 of *L. crispatus* supernatant on Hela cells was significantly lower than its IC50 on HNCF cells revealing that it had higher inhibitory effect on tumoral cervical cells compared with normal cervical cells. All other lactobacilli supernatants had similar effects on either cell lines or even lower cytotoxic effect on tumoral cells. Cell line behaviors were similar in trypan blue staining and MTT assay.

## 5. Discussion

The health in women’s urogenital tracts is largely dependent on the function of the vaginal microflora. Lactobacilli have been indicated as the dominate microorganism of the normal vaginal flora. They play an important role in protecting against pathogens invasion or overgrowth by production of hydrogen peroxide, bacteriocins, and lactic acid (27). In the current study, PCR methods were used to compare the spread of Lactobacillus species known in the vaginal flora of healthy women and BV infected women. Molecular methods (such as PCR) compared with biochemical methods may reduce observer-dependent errors and provide more direct and detailed results (14). In the past, vaginal flora of healthy women was believed to be dominated by *L. acidophilus* and *L. fermentum*, followed by *L. brevis*, *L. jensenii*, *L. casei*, and other species (28).But recent studies using molecular methods indicated that *L. crispatus*, *L. iners*, and *L. jensenii* were the most common lactobacilli isolates of healthy vaginal flora (9). There are certain similarities between current study results with the results from the recent studies and it was found that *L. crispatus*, *L. gasseri*, *L. iners*, *L. jensenii*, *L. acidophilus* and *L. rhamnosus* were the most common species. The current study found that the vaginal lactobacilli composition of Iranian women had clear difference with some other communities. For example, Lactobacillus species present in healthy vagina of Indian women were *L. reuteri* (32.5%), *L. fermentum* (25%), and *L. salivarius* (16.25%) (12). Importantly, there were also significant differences in the abundance of common lactobacilli among women of different racial groups. .For instance, the amount of *L. crispatus*, *L. gasseri*, *L. jensenii*, and *L. iners* in the healthy Chinese women were 91%, 67%, 43% and 75%, respectively (14)but Iranian women’s abundance of these lactobacilli were 66.7%, 29.6%, 29.6% and 55%, respectively.Furthermore, it is widely accepted that the microbial balance between lactobacilli as the dominating flora and other anaerobes can frequently result in the infectious condition of bacterial vaginosis (8). Thus, the lactobacillus species of the healthy human vagina has to be compared with those of BV infected ones to define the protective lactobacilli. A study in South Africa showed that *L. crispatus* isolates were almost equally distributed between individuals with and without BV and *L. jensenii* isolates were significantly reduced in women infected with BV (11). But, another study by Yan et al. in china revealed that the quantities of *L. crispatus*, *L. gasseri* and *L. iners* were higher in healthy women but the *L. jensenii* did not show any significant difference (14). Verstraelen et al. revealed that in Belgian women, *L. crispatus* promoted the stability of the normal vaginal microflora while *L. gasseri* and/or *L. iners* predisposed to some extent to the occurrence of abnormal vaginal microflora (29). Another study in Japan showed that the presence of *L. iners* may be correlated with vaginal colonization by BV-related bacteria (30). The similarity of the current study results with these data is that the current study confirmed that *L. crispatus* and *L. jensenii* had higher quantities in healthy women and also *L. iners* did not have any specific role in preventing bacterial infection. Another aim of the current study was to examine the effect of different lactobacilli on the cervical cancer. Many studies investigated Lactobacillus effects on cell lines, for example Russo et al. showed that lactobacilli can exert anti-proliferative effects on the gastric epithelium (16). Also Peluso et al. indicated that *L. paracasei* subsp. paracasei inhibits T-cell growth, and it can interfere with T-cell-driven immune responses (31). The current study compared the effect of 3 vaginal lactobacilli with 3 commercial vaginal probiotics to determine whether anti-cancer effects of lactobacilli play a role in their selection as vaginal probiotics. It is revealed that commercial probiotics do not have more cytoxicity effect on tumoral cervical cells compared to normal cells. Overall, the only lactobacillus species which had more inhibitory effect on tumoral cells was *L. crispatus* strain SJ-3C-US and it can be proposed as a vaginal probiotic, although further studies should be implemented. Although Amin et al. identified 3 rare vaginal lactobaciili of healthy Iranian women (32), the current study analyzed 12 common and rare lactobacilli in healthy and BV infected women. Also, they didn’t use Hey grading criteria as a standard method for grading the specimens. Thus this is the first-ever study of vaginal lactobacilli from healthy and BV infected Iranian women. The study determined that there were similarities and differences between the composition of vaginal lactobacilli detected in our country and those from distinctly different environments. It was hypothesized that these differences may be the cause of differences in the susceptibility of women to BV and various vaginal infections.
